# A Spinal Cord Sarcoma With *EWSR1::WT1* Fusion Exhibited Ewing Sarcoma‐Like Pattern Rather Than Desmoplastic Small Round Cell Tumor

**DOI:** 10.1111/pin.70181

**Published:** 2026-09-27

**Authors:** Chuqian Zeng, Yishan Wang

**Affiliations:** ^1^ Department of Pathology The First Affiliated Hospital of Jinan University Guangzhou China

**Keywords:** desmoplastic small round cell tumor, Ewing sarcoma‐like, *EWSR1::WT1* fusion, next generation sequencing, spinal cord sarcoma

## Abstract

We received a case of a 22 years old female, with a spinal cord sarcoma. The tumor exhibited a morphology similar to that of a small blue round cell tumor, lack of desmoplastic stroma. Immunohistochemical analysis demonstrated diffusely membranal expression of CD99 and cytoplasmic expression of Desmin, but was negative of NKX2.2. Fluorescence in situ hybridization indicated the EWSR1 gene rearrangement. Next generation sequencing (NGS) identified a gene fusion of *EWSR1(E9)::WT1(E8)*. The fusion gene was confirmed by RNA NGS as well. There was a challenge on diagnosis whether should it be diagnosed as Ewing sarcoma‐like tumor or DSRCT. Conclusion: The *EWSR1::WT1* fusion tumor has a wide spectrum of diseases. The morphology may be associate with specific gene breakage site, or/and tissue specificity. Final diagnosis should be rendered based on comprehensive correlation of clinical presentation, morphology, immunophenotype, and molecular finding.

## Graphical abstract

We received a case of a 22 years old female, with a spinal cord sarcoma. The tumor consisted of small blue round cells, lack of desmoplastic stroma, with positive expression of CD99 and Desmin in IHC, negative expression of NKX2.2 in IHC, separative signals of *EWSR1* gene probe in FISH, gene fusion of *EWSR1(E9)::WT1(E8)* through RNA and DNA NGS. There was a challenge on diagnosis whether should it be diagnosed as Ewing sarcoma‐like tumor or DSRCT.

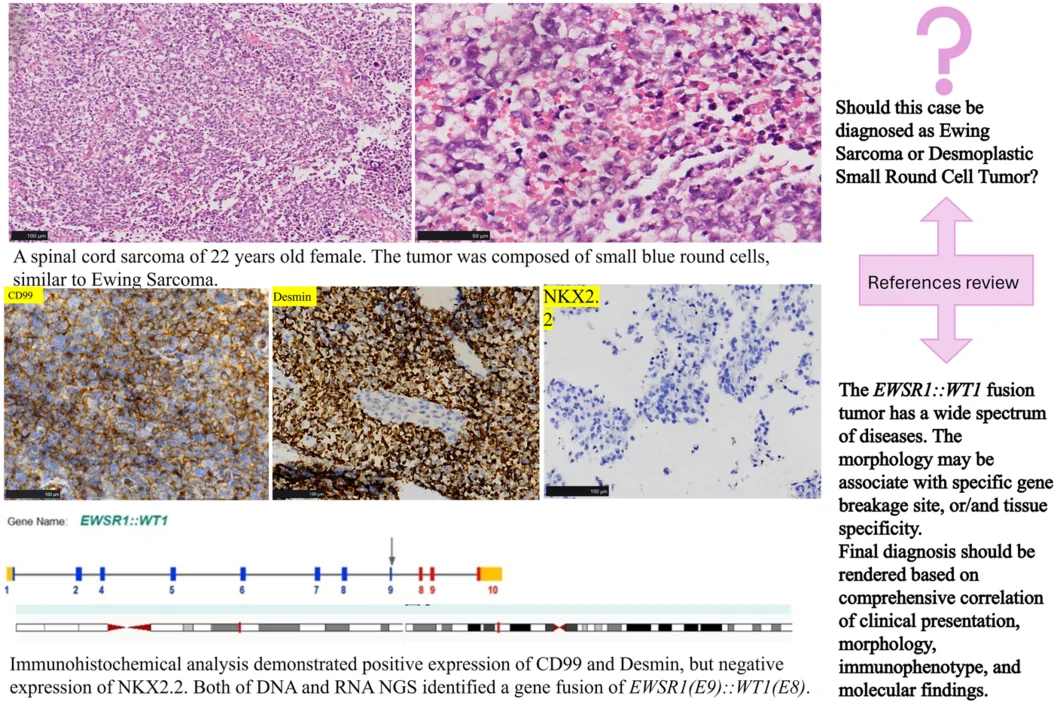

## 1 Introduction

*EWSR1::WT1* fusion is regarded as characteristic gene fusion of desmoplastic small round cell tumor (DSRCT). According to the 5th WHO classification of soft tissue and bone tumors, DSRCT is a malignant mesenchymal neoplasm composed of small round cells associated with prominent stromal desmoplasia, polyphenotypic differentiation, and *EWSR1::WT1* fusion. But it lacks of secondary mutations but a significant number of copy number alterations. It is hypothesized that the N‐terminal domain of *EWSR1* acts as a transcriptional activator, while the zinc finger domains of *WT1* binds regulatory elements in target genes [1]. Recent analyzes of mutational profiles revealed the deregulation of genes related to immune response, epithelial–mesenchymal transition (EMT), and the DNA damage response (DDR) in DSRCT [2, 3].

In the 5th WHO classification of soft tissue and bone tumors, undifferentiated small round cell tumor comprises Ewing sarcoma, round cell sarcoma with *EWSR1*‐non‐*ETS* fusion, *CIC*‐rearranged sarcoma, sarcoma with *BCOR* genetic alterations [4]. Ewing sarcoma is a small cell sarcoma with gene fusions involving one member of the *FET* family (usually *EWSR1*) and a member of the *ETS* family [4]. In this category, small round cell sarcoma with *EWSR1‐*non‐*ETS* fusion include the *EWSR1::NFATC2*‐fusion and the *EWSR1::PATZ1*‐fusion sarcoma. Pitfalls in ES diagnosis include aberrant expression of immunohistochemical markers. Neuroendocrine markers and/or desmin are rarely seen, but keratin expression may occur in up to 25% of cases [5]. It was discovered that *EWSR1* is rearranged in clear cell sarcoma, angiomatoid fibrous histiocytoma, extra‐skeletal myxoid chondrosarcoma and hyalinising clear cell carcinoma, among others [6]. In our case, the spinal cord sarcoma exhibited Ewing sarcoma‐like pattern lack of desmoplastic stromal, with *EWSR1::WT1* fusion. Cordier F reported a case of an undifferentiated sarcoma of the bone with a round to epithelioid cell phenotype harboring a novel *EWSR1‐SSX*2 fusion. The author thought it could be a new member belonging to the heterogeneous group of round cell sarcomas with *EWSR1*‐non‐*ETS* fusion [7]. *EWSR1* fusions are present in tumor types that show morphological overlap with one another. Specific *EWSR1* fusion genes has led to some confusion and controversy in the tumor classification system [6]. The reports reminded us that our case might be a situation like this, because our case displayed a pattern similar to Ewing sarcoma rather than DSRCT, and strongly co‐expressed CD99 and desmin.

## 2 Case Description

**Figure 1:**
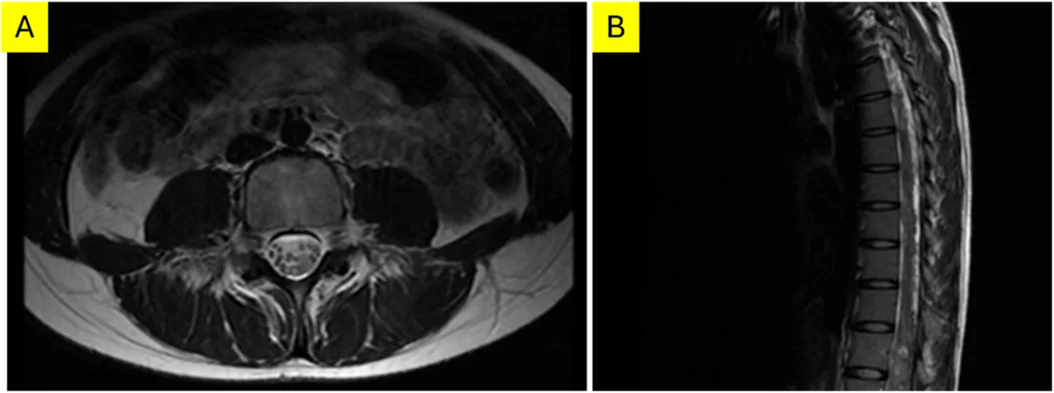
It demonstrated heterogeneous hyperintensity on sagittal and axial T2WI with multiple internal cystic areas, and obscure boundary.

We received a case of a 22 years old female, suffering from progressive weakness and limping in both lower extremities, as well as low back pain in More than twenty days. She had accepted magnetic resonance imaging. The spinal cord was thickened, showed irregular long T1 and T2 signal shadows at the T12‐L1 level, multiple small cystic changes within it, measuring approximately 5 cm long. Enhanced scanning revealed uneven enhancement (as shown in Figure 1). In addition, abnormal fusiform signal shadows were observed at the T5/6 and T8/9 vertebrae. Multiple small nodular with abnormal signal shadows could be seen on the surface of the dura mater and spinal cord, and uneven enhancement while enhanced scanning.

**Figure 2:**
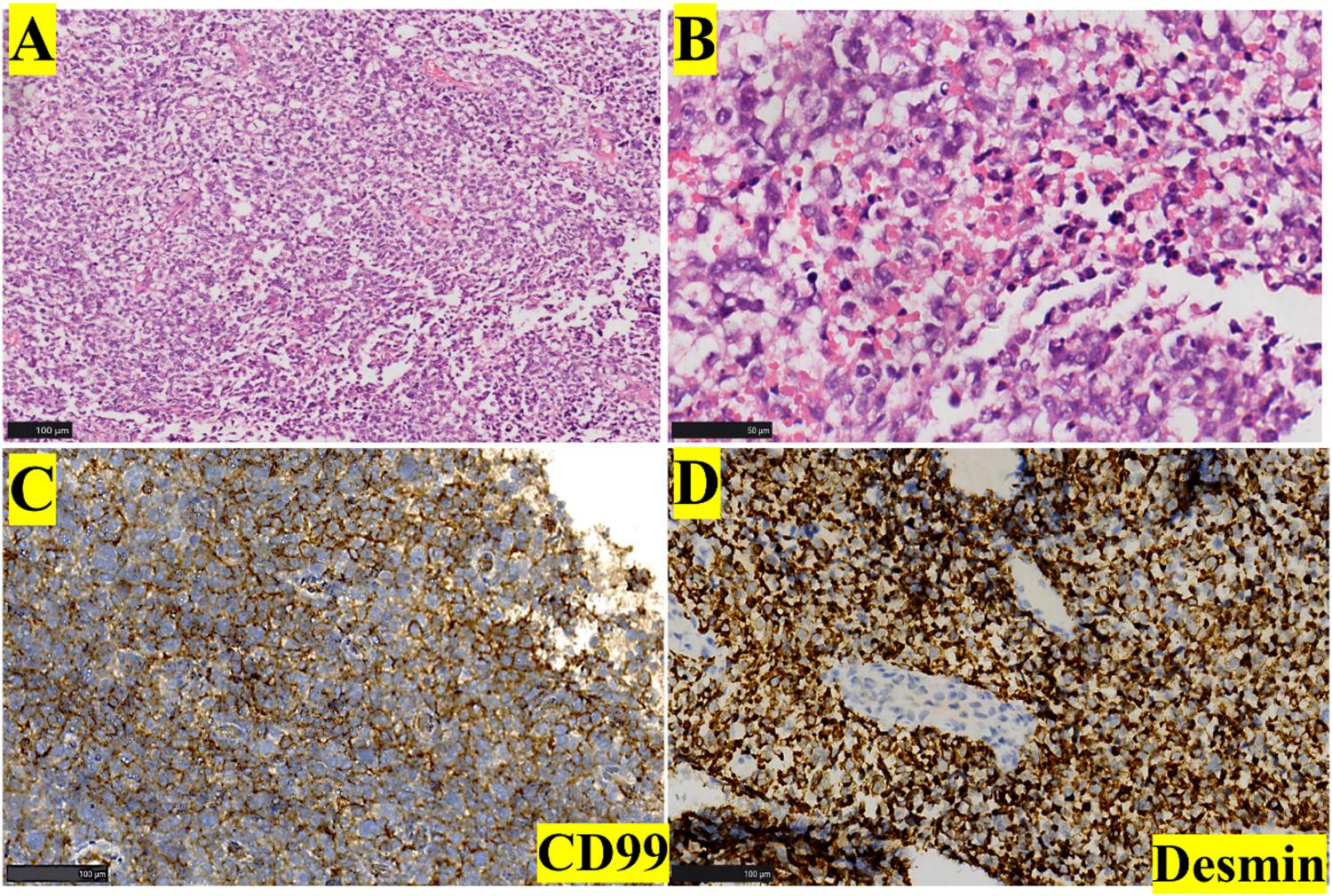
(A) The tumor exhibited a diffuse growth pattern. There was few fibrous stromal. H&E, x100. (B) The neoplasm consisted of small round cells demonstrating morphological uniformity. The tumor cells displayed scant cytoplasm, resulting in a high nuclear‐to‐cytoplasmic ratio. Nuclei were irregular with finely stippled (‘powdery’) chromatin, inconspicuous nucleoli. Brisk mitotic activity (8～18 mitoses/10 HPF) were found. H&E, x400. (C) CD99 was diffusely positive on cytomembrane. IHC x200. (D) Cytoplasmic expression of Desmin. However, part of tumor cells expressed Desmin with dot‐like pattern. IHC x200.

She underwent a tumor resection for pathological examination. Microscopically, the tumor exhibited a diffuse growth pattern with focal necrosis. The neoplasm consisted of small round cells demonstrating morphological uniformity (as shown in Figure 2A). The tumor cells displayed scant cytoplasm, resulting in a high nuclear‐to‐cytoplasmic ratio. Nuclei were irregular with finely stippled (‘powdery’) chromatin, inconspicuous nucleoli (as shown in Figure 2B). Brisk mitotic activity (8～18 mitoses/10 HPF) was found. There was few fibrous stromal.

**Figure 3:**
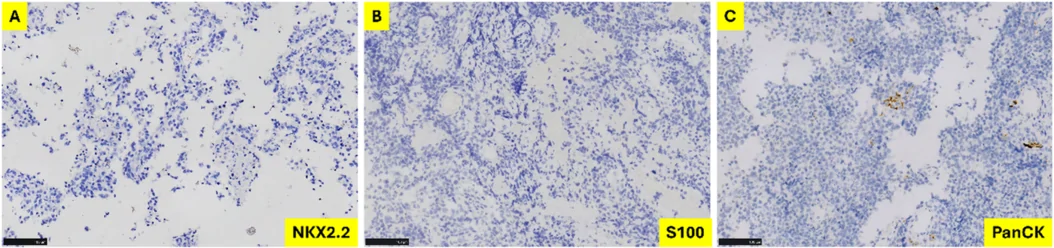
(A) NKX2.2 was negative. IHC x200. (B) S100 was negative. IHC x200. (C) Focal weak expression was observed of Pan‐CK. IHC x200.

Immunohistochemical analysis demonstrated diffusely membranal expression of CD99 and cytoplasmic expression of Desmin (as shown in Figure 2C,D). However, part of tumor cells expressed Desmin with dot‐like pattern. P53 is diffusely positive. Focal weak expression was observed for Pan‐CK (as shown in Figure 3C), FLI‐1, BCOR, MyoD1, Synaptophysin, EMA. Normal expression was detected for INI‐1, SMARCA4, H3k27me3 without any loss. The tumor was negative for NKX2.2 (as shown in Figure 3A), C‐myc, S‐100 (as shown in Figure 3B), Sox10, SS18, P63, WT1(N‐terminal), Chromogranin A, Myogenin, SSTR2, olig2, GFAP, CD3, CD20, CD30, H3K27M, SALL4, OCT3/4, HMB45, Melan‐A, Branchyury and CD34. The Ki‐67 proliferative index was approximately 50%.

**Figure 4:**
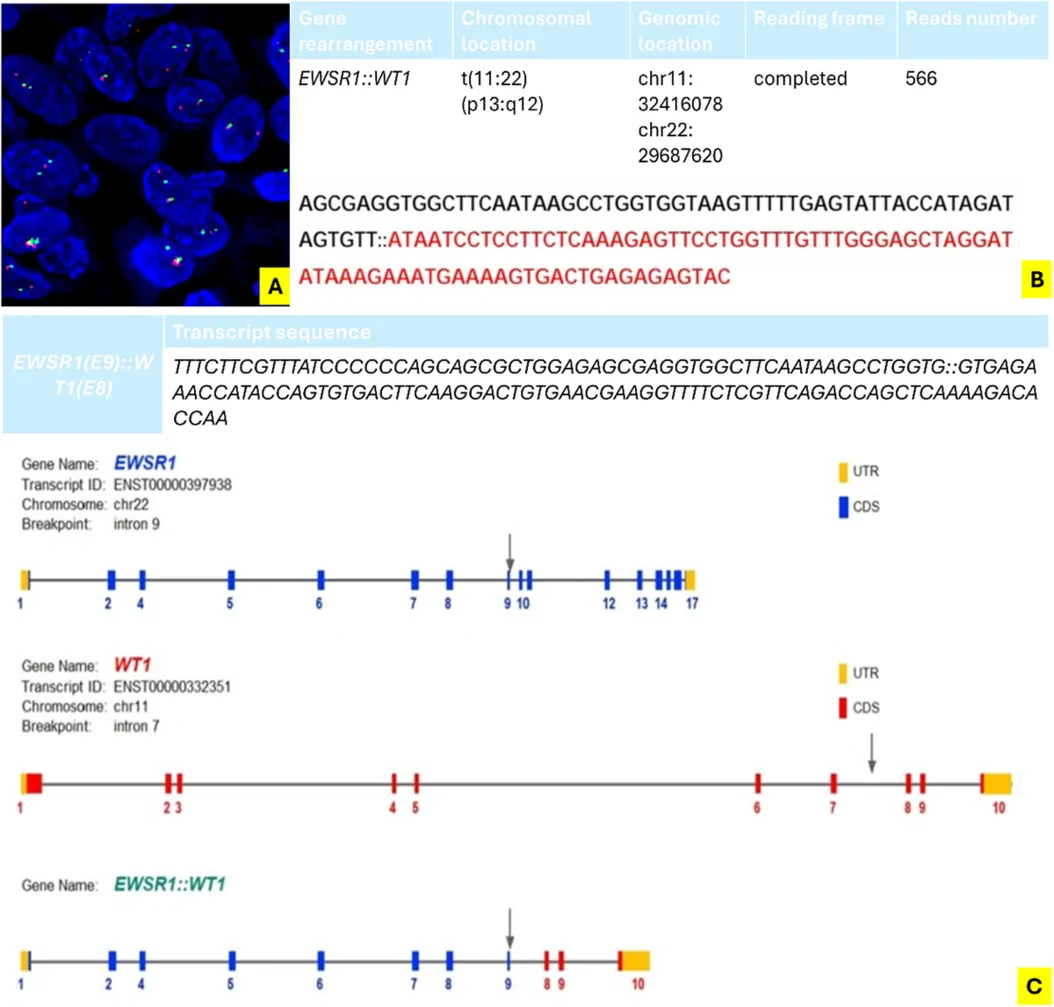
(A) Fluorescence in situ hybridization showed red‐green split signals of *EWSR1* Break Apart Probe. (B) Next generation sequencing (NGS) identified a gene fusion of *EWSR1::WT1* that fused exon 9 of *EWSR1* (chr22:29687620) with exon 8 of *WT1* (chr11:32416078). (C) RNA NGS detected the transcription product of *EWSR1(E9)::WTI(E8)* fusion gene.

Fluorescence in situ hybridization (EWSR1 Break Apart Probe, Ibp) showed split signals, which indicated the EWSR1 gene rearrangement (as shown in Figure 4A).

Next generation sequencing (NGS) identified a gene fusion of *EWSR1::WT1* that fused exon 9 of *EWSR1* (chr22:29687620) with exon 8 of *WT1* (chr11:32416078) (as shown in Figure 4B). The fusion gene was confirmed by RNA NGS with the MAP Solid Tumor Fusion Gene Detection Kit on lllumina NovaSeq. 6000 (as shown in Figure 4C). In addition to the fusion gene, it was detected *TP53* mutation, LOH of *CDKN2A* and *TP53*, homozygous deletion of *PTCH1* and *FANCC*. Analysis of whole‐genome copy number variation (CNV) and loss of heterozygosity (LOH) revealed copy number gains in the following chromosomal regions: 4q, 5q35.2 → q35.3, 8p23.3 → q13.2, 8q24.3, 17q, and Xq11.2 → q13.1; copy number losses in the following regions: 3p, 4p, chromosome 9 (with deletions of CDKN2A/B, FANCC, and PTCH1), 14q11.2 → q24.3, 16q11.2 → q21, chromosome 17 (with TP53 deletion), 21q11.2 → q21.3, and Xq13.2 → q28; and CN‐LOH in the following regions: 8q13.3 → q21.3, chromosome 13, and 16q22.1 → q24.3.

## 3 Discussion

Combined with morphology and immunohistochemical analysis, we had excluded several diagnoses, included in poorly differentiated synovial sarcoma, alveolar rhabdomyosarcoma with solid pattern, mesenchymal chondrosarcoma, neuroendocrine neoplasm and lymphoma. Although the tumor was composed of small round cells with scarce mesenchyme, a pattern similar to Ewing sarcoma. DNA and RNA NGS detected the fusion gene of *EWSR1::WTI*, and immunohistochemistry showed negative expression of the NKX2.2 antibody; these findings do not support a diagnosis of Ewing sarcoma. It was a big challenge for us to diagnosed as desmoplastic small round cell tumor (DSRCT) or Round cell sarcoma with *EWSR1*‐non‐*ETS* fusion.

Referred to references, many cases which demonstrated unlike the classical morphology of DSRCT were reported (as shown in Table 1). These cases harbored *EWSR1::WT1* fusion, but lack of, or few of desmoplastic stroma. The locations of lesions included abdominal cavity, axillary soft tissue, retroperitoneal soft tissue, female genital tract, cauda equina. The patients were 12–85 years old, respectively. They showed a wide morphological spectrum, consisted of epithelioid cells, spindle shaped cells, small round cells [8, 9, 10, 11, 12, 13, 14, 15, 16]. Some of the authors considered these cases as a variant of DSRCT with atypical morphology. Others thought of a new entity or subsets of discrete entities with *EWSR1::WTI* fusion, rather than DSRCT, according to morphology [8, 9, 10, 11, 12, 13, 14, 15, 16].

**Table 1:** Clinicopathological, immunohistochemical, and molecular features of reported EWSR1–WT1 fusion‐associated tumors which lack/ few of desmoplastic stroma.

Author	Age/sex	Location	Histopathology	IHC	Gene alteration	Outcome
Ali et al.^8^	16/F	Abdominal cavity	Uniform small round cells arranged in sheet‐like architecture	Diffused expression: WT1 (carboxy terminal); focal expression: neuron‐specific enolase (NSE)	N/A	N/A
Felix et al.^9^	26/M	Peritoneum of the left lower abdomen	Composed of monotonous small round to polygonal cells growing in small, confluent nests, trabeculae, and strands	Diffuse and strong cytoplasmic expression of Desmin	Fusion that fused exon 7 of EWSR1 with exon 8 of WT1	No recurrent 10months after resection
Kihara et al.^10^	51/M	Abdominal cavity	Small round cells arranged in solid pattern	Diffused expression: Desmin, BCL2, CD99; focal expression: AE1/AE3, CAM5.2, NSE, Syn, SMA, actin	Fusion of EWSR1 exon 7 and WT1 exon 8	N/A
AGNES S et al.^11^	15/F	Retroperitoneal mass	Small primitive cells forming well‐defined nests	Diffused expression: Desmin, vimentin; focal expression: keratin, NSE, and CD99.	Fusion of EWSR1 exon 9 and WT1 exon 8	N/A
Magro et al.^12^	12/M	Abdominal cavity	Spindle cells arranged into short, intersecting fascicles	Expression: vimentin, desmin, WT‐1 (C‐terminus antibodies) and EMA	Fusion of EWSR1 exon 9 and WT1 exon 7	Died 9 months after the diagnosis
Alaggio et al.^13^	Adolescent	Abdominal cavity	Spindle cells arranged in sheets or in fascicles	Expression: Cytokeratins SMA, desmin, and actin	RT‐PCR analysis showed an EWS‐WT1 fusion transcript	Case1 alived at 3 years after diagnosis
						Case2 alived at 13 years after diagnosis
Zhang et al.^14^	17/F	Abdominal cavity	Small round cells lining microcystic structures, forming pseudoacini and trabeculae and cords.	Expression: vimentin, desmin, SMA, Syn, NSE, Bcl‐2 and WT1	N/A	N/A
Schoolmeester et al.^15^	46/F	Cervix	Mostly were spindled cells arranged in a mixture of sheet‐like and fascicular architecture	Diffused expression: Desmin; focal expression: AE1/AE3	Fusion of EWSR1 exon 9 and WT1 exon 8	No recurrent 9 months after diagnosis.
	30/F	Large mass involving left ovary, myometrium, retroperitoneum, peritoneum, uterine serosa	Composed of sheets and fascicles of cells with cystic change	Diffused expression: Desmin; focal expression: AE1/AE4, SMA	Fusion of EWSR1 exon 10 and WT1 exon 7, in addition, WT1 had a 12 bp in‐frame deletion.	Lymph node metastasis 7 months after diagnosis
	20/F	Mass centered in the left vaginal fornix	Composed of predominantly fascicular of spindle cells	Diffused expression: Desmin, WT1; focal expression: AE1/AE5, SMA	Fusion of EWSR1 exon 9 and WT1 exon 9	Alived at three and a half years after diagnosis
Ud Din et al.^16^	34/M	L4 nerve root	Composed of small round cells, arranged in nests and cords, focally show rosette‐like structures	Diffused expression: Desmin, SMA; focal expression: CD99, NeuN,	A fusion sequence between exon 7 of EWSR1 and exon 8 of WT1	Alived a 9 months after surgery

Abbreviations: AE1/AE3, cytokeratin AE1/AE3; BCL2, B‐cell lymphoma 2; CAM5.2, cytokeratin CAM5.2; CD99, cluster of differentiation 99; EMA, epithelial membrane antigen; EWSR1, Ewing sarcoma breakpoint region 1; IHC, immunohistochemistry; N/A, not available; NSE, neuron‐specific enolase; RT‐PCR, reverse transcription‐polymerase chain reaction; SMA, smooth muscle actin; Syn, synaptophysin; WT1, Wilms tumor 1.

The tumor presented in this case was not DSRCT for several reasons. DSRCT mainly develops in abdominal cavity. In our case, the female patient developed tumor in spinal cord, any desmoplastic stroma can be seen under microscope. Due to microscopic morphology and lesion location, we preferred to consider our case as small cell sarcoma with *EWSR1*‐non‐*ETS* fusion, even it was *EWSR1::WT1* gene fusion. Interestingly, it co‐expressed CD99 and Desmin strongly.

Ultimately, this study lends strong support to the notion that even the specific *EWSR1::WT1* fusion of DSRCT occurs in other tumor types and therefore is not strictly disease‐defining. It probably encompasses tumor entities with different clinical, morphologic, and immunohistochemical characteristics. *EWSR1::WT1* chimeras have been described in which exons 7–11 of the *EWSR1* gene are fused to exon 8–10 of the *WT1* gene. Each of these fusion proteins may include or lack a Lys‐Thr‐Ser (KTS) insert located between zinc fingers 3 and 4 of the *WT1* portion of the chimera [1, 2, 17, 18, 19, 20]. The alternative splicing different isoforms of the fusion protein maybe an essential clue accounts for morphological diversity of non‐DSRCT *EWSR1::WT1* fusion tumor.

We noted a case similar to ours, which AGNES S. CHAN reported in 1999 [11]. A 15‐year‐old female presented with a retroperitoneal mass. The patient underwent needle biopsy of the mass. The tumor was composed of small, uniform cells with hyperchromatic nuclei without identifiable nucleoli. Tumor cells showed no differentiation or particular architectural arrangements, without desmoplastic reaction. The tumor cells stained strongly for desmin, and focally for keratin, neuron‐specific enolase, and CD99. No staining for WT1 was noted. RT‐PCR sequencing showed an in‐frame fusion of *EWSR1* exon 9 to *WT1* exon 8. The same gene breakage site resulted in similar fusion protein. It may indicate similar tumor‐driving mechanism, and then show a similar microscopical pattern. The alternative splicing different isoforms of the fusion protein is responsible for heterogeneity of *EWSR1::WT1* fusion tumor.

Schoolmeester J et al. reported two cases occurring in female genital tract. Both of them had *EWSR1(E9)::WT1(E8)* gene fusion like ours [15]. The neoplastic cells were spindled for the most part, were smaller and more epithelioid in some portions, arranging in mainly sheets and fascicles, occasionally nested and corded architecture. The morphology was not similar to ours. We supposed that the distinction on morphology may be associate with tissue specificity, in addition to specific gene breakage site.

An *EWSR1‐WT1* fusion alone is not sufficient for a reliable diagnosis of DSRCT, since the *EWSR1::WT1* fusion tumor has a wide spectrum of diseases. Final diagnosis should be rendered cautiously and appropriately based on comprehensive correlation of clinical presentation, morphology, immunophenotype, and molecular findings. It is advisable to include comments addressing prognostic stratification and follow‐up management recommendations when applicable.

## Author Contributions

**Chuqian Zeng:** conceptualization, study design, data analysis, methodology, visualization, software, writing, review, supervision. **Yishan Wang:** data Acquisition, performed experiments, collected data, review. All authors have read and approved the final manuscript and agree to be accountable for all aspects of this work, ensuring that questions related to the accuracy or integrity of any part of the work are appropriately investigated and resolved.

## Funding

The authors have nothing to report.

## Consent

Written informed consent was obtained from the patient included in the study.

## Conflicts of Interest

The authors declare no conflicts of interest.

## Data Availability

Data sharing not applicable to this article as no datasets were generated or analyzed during the current study.

## References

[1] L. Marcelis and A. L. Folpe , “‘Putting the Cart before the Horse’: An Update on Promiscuous Gene Fusions in Soft Tissue Tumors,” Virchows Archiv 486, no. 5 (2025): 905–921. 10.1007/s00428-025-04099-1 40205020

[2] L. Anastasia and M. S. Janet , “Desmoplastic Small Round Cell Tumor (DSRCT): Emerging Therapeutic Targets and Future Directions for Potential Therapies,” Expert Opinion on Therapeutic Targets 24, no. 4 (2020): 281–285. 10.1080/14728222.2020.1738392 32125905

[3] A. Devecchi , L. De Cecco , M. Dugo , et al., “The Genomics of Desmoplastic Small Round Cell Tumor Reveals the Deregulation of Genes Related to Dna Damage Response, Epithelial‐Mesenchymal Transition, and Immune Response,” Cancer Communications 38, no. 1 (2018): 70. 10.1186/s40880-018-0339-3 PMC6260689 30486883

[4] C. A. Dehner , A. J. Lazar , and J. S. A. Chrisinger , “Updates on Who Classification for Small Round Cell Tumors: Ewing Sarcoma vs. Everything Else,” Human Pathology 147 (2024): 101–113. 10.1016/j.humpath.2024.01.007 38280658

[5] J. Davis and E. Rudzinski , “Small Round Blue Cell Sarcoma Other Than Ewing Sarcoma: What Should An Oncologist Know?,” Current Treatment Options in Oncology 21, no. 11 (2020): 90. 10.1007/s11864-020-00785-1 32875423

[6] T. Emily Anne and P. David James , “Ewsr1: The Promiscuous King of Mesenchymal Neoplasia,” Journal of Clinical Pathology 77, no. 11 (2024): 721–725. 10.1136/jcp-2023-208867 39209444

[7] F. Cordier , J. Van der Meulen , B. Van Gaever , et al., “Undifferentiated Sarcoma of Bone With a Round to Epithelioid Cell Phenotype Harboring a Novel EWSR1‐SSX2 Fusion Identified by RNA‐Based Next‐Generation Sequencing,” Genes, Chromosomes & Cancer 61, no. 1 (2022): 44–49. 10.1002/gcc.22999 34538011

[8] A. Ali , M. Mohamed , J. Chisholm , and K. Thway , “Solid‐Pattern Desmoplastic Small Round Cell Tumor,” International Journal of Surgical Pathology 25, no. 2 (2017): 158–161. 10.1177/1066896916660198 27431755

[9] K. Felix , D. Wolfgang , F. Alois , et al., “Expanding the Spectrum of Tumors With ewsr::Wt1 Gene Fusion by a Peritoneal Case That Is Not a Desmoplastic Small Round Cell Tumor,” Modern Pathology 36, no. 5 (2023): 100156. 10.1016/j.modpat.2023.100156 36918056

[10] A. Kihara , K. Takahashi , A. Ishikawa , et al., “Desmoplastic Small Round Cell Tumor Showing Solid Proliferation With Limited Desmoplasia and Confusing Immunohistochemical Findings: An Autopsy Report,” Medical Molecular Morphology 53, no. 3 (2020): 177–182. 10.1007/s00795-019-00242-5 31907620

[11] A. S. Chan , S. MacNeill , P. Thorner , J. Squire , and M. Zielenska , “Variant ews‐wt1 Chimeric Product in the Desmoplastic Small Round Cell Tumor,” Pediatric and Developmental Pathology 2, no. 2 (1999): 188–192. 10.1007/s100249900108 9949226

[12] G. Magro , G. Broggi , A. Zin , et al., “Desmoplastic Small Round Cell Tumor With ‘Pure’ Spindle Cell Morphology and Novel EWS‐WT1 Fusion Transcript: Expanding the Morphological and Molecular Spectrum of This Rare Entity,” Diagnostics 11, no. 3 (2021): 10.3390/diagnostics11030545. 10.3390/diagnostics11030545 PMC8003219 33803887

[13] R. Alaggio , A. Rosolen , F. Sartori , et al., “Spindle Cell Tumor With EWS‐WT1 Transcript and a Favorable Clinical Course: A Variant of DSCT, a Variant of Leiomyosarcoma, or a New Entity? Report of 2 Pediatric Cases,” American Journal of Surgical Pathology 31, no. 3 (2007): 454–459. 10.1097/01.pas.0000213375.02171.43 17325488

[14] J. Zhang , J. Dalton , and C. Fuller , “Epithelial Marker‐Negative Desmoplastic Small Round Cell Tumor With Atypical Morphology: Definitive Classification by Fluorescence In Situ Hybridization,” Archives of Pathology & Laboratory Medicine 131, no. 4 (2007): 646–649. 10.5858/2007-131-646-EMDSRC 17425400

[15] J. K. Schoolmeester , A. L. Folpe , A. A. Nair , et al., “EWSR1‐WT1 Gene Fusions in Neoplasms Other Than Desmoplastic Small Round Cell Tumor: A Report of Three Unusual Tumors Involving the Female Genital Tract and Review of the Literature,” Modern Pathology 34, no. 10 (2021): 1912–1920. 10.1038/s41379-021-00843-5 34099870

[16] N. Ud Din , M. Pekmezci , G. Javed , et al., “Low‐Grade Small Round Cell Tumor of the Cauda Equina With EWSR1‐WT1 Fusion and Indolent Clinical Course,” Human Pathology 46, no. 1 (2015): 153–158. 10.1016/j.humpath.2014.09.015 25454478

[17] M. W. Laura , P. Raul , L. Pauline , et al., “Ewsr1::Wt1 Fusions in Neoplasms Other Than Conventional Desmoplastic Small Round Cell Tumor: Three Tumors Occurring Outside the Female Genital Tract,” Modern Pathology 37, no. 3 (2024): 100418. 10.1016/j.modpat.2023.100418 38158126

[18] F. Ina , K. Scott , G. Tova , et al., “Transcriptional Regulation of Igf‐I Receptor Gene Expression by Novel Isoforms of the ews‐wt1 Fusion Protein,” Oncogene 21, no. 12 (2002): 1890–1898. 10.1038/sj.onc.1205042 11896622

[19] E. K. Slotkin , A. S. Bowman , M. F. Levine , et al., “Comprehensive Molecular Profiling of Desmoplastic Small Round Cell Tumor,” Molecular Cancer Research 19, no. 7 (2021): 1146–1155. 10.1158/1541-7786.MCR-20-0722 PMC8293793 33753552

[20] P. Dundr , J. Drozenová , R. Matěj , et al., “Desmoplastic Small Round Cell Tumor of the Uterus: A Report of Molecularly Confirmed Case With EWSR1‐WT1 Fusion,” Diagnostics 12, no. 5 (2022): 1184. 10.3390/diagnostics12051184 PMC9140206 35626339

